# Fungemia Due to Saprochaete capitata in a Non-Neutropenic Critically Ill Patient

**DOI:** 10.7759/cureus.51147

**Published:** 2023-12-27

**Authors:** Tala N Mawad, Rakan A Alfaifi, Othman M Almazyed, Rand A Alhumaidi, Abdulaziz M Alsubaie

**Affiliations:** 1 Infectious Diseases, King Saud University, Riyadh, SAU; 2 Internal Medicine, King Saud University, Riyadh, SAU

**Keywords:** magnusiomyces capitatus, a case report, non-candida yeast, disseminated fungemia, antifungal therapy, non-neutropenic patient, saprochaete capitata

## Abstract

*Saprochaete capitata* is an uncommon yeast species; its impact on non-neutropenic patients appears to be on the rise. We describe a case of *S. capitata* fungemia in a critically ill end-stage kidney disease (ESKD) patient on peritoneal dialysis. The patient presented with mesenteric ischemia and underwent several laparotomies during hospitalization. His hospital stay was complicated as fungemia developed and spread to multiple sites, which resulted in severe complications and ultimately led to fatal outcomes. *S. capitata*'s diagnostic delay is a concern, but matrix-assisted laser desorption/Ionization time-of-flight (MALDI-TOF) mass spectrometry may help provide accurate identification. Our case highlights the need for prompt diagnosis and tailored antifungal therapy, especially when managing this challenging infection in immunocompromised patients.

## Introduction

*Saprochaete capitata* is a rather unusual cause of nosocomial illness. The majority of cases occur in immunocompromised, neutropenic, or critically ill patients in the intensive care unit (ICU). Over the past decade, the frequency of uncommon fungal infections in non-neutropenic patients increased exponentially [1]. The clinical picture is similar to that of invasive candidiasis, but empirical IV antifungal treatment is ineffective. As a result, rapid molecular identification and prompt beginning of suitable antifungal medication are required. However, the susceptibility patterns to common antifungal medications vary, and there is a scarcity of convincing clinical data to define the optimum therapeutic approach.

## Case presentation

A 60-year-old man, a known end-stage kidney disease (ESKD) patient on peritoneal dialysis with poorly controlled type 2 diabetes mellitus and hypertension, presented to the emergency department (ED) with loss of consciousness and shortness of breath after his second cycle of dialysis. Examination revealed a wide pulse pressure (154/43 mmHg) and tachypnea (28 breaths per minute). On arrival to ED, the patient was unresponsive (Glasgow Coma Scale (GCS) 7) and hypoxic on 15 L of oxygen with a distended abdomen and crackles audible over the right lung base. Further investigations are shown in Table 1.

**Table 1:** Lab values obtained on admission HCO_3_: bicarbonate, ALT: alanine aminotransferase, AST: aspartate aminotransferase; INR: international normalized ratio; APPT: activated partial thromboplastin clotting time; PT: prothrombin time

Lab view	Patient values	Reference range
WBC	16.3 × 10^9^/L	4-11 × 10^9^/L
Hemoglobin	55 gm\L	130-180 gm\L
Arterial blood gas, pH	6.89	7.340-7.450
HCO_3_	6.7 mmol\L	22-29 mmol\L
Creatinine	1082 mcmol\L	62-115 mcmol\L
Lactate	22.4 mmol/L	0.5-2.2 mmol/L
ALT	20.4	0-41 unit/L
AST	63.9	0-40 unit/L
INR	1.83	0.8-1.30 seconds
APPT	44.6	25.7-39.5 seconds
PT	23.7	12.10 -15.7 seconds

**Figure 1:**
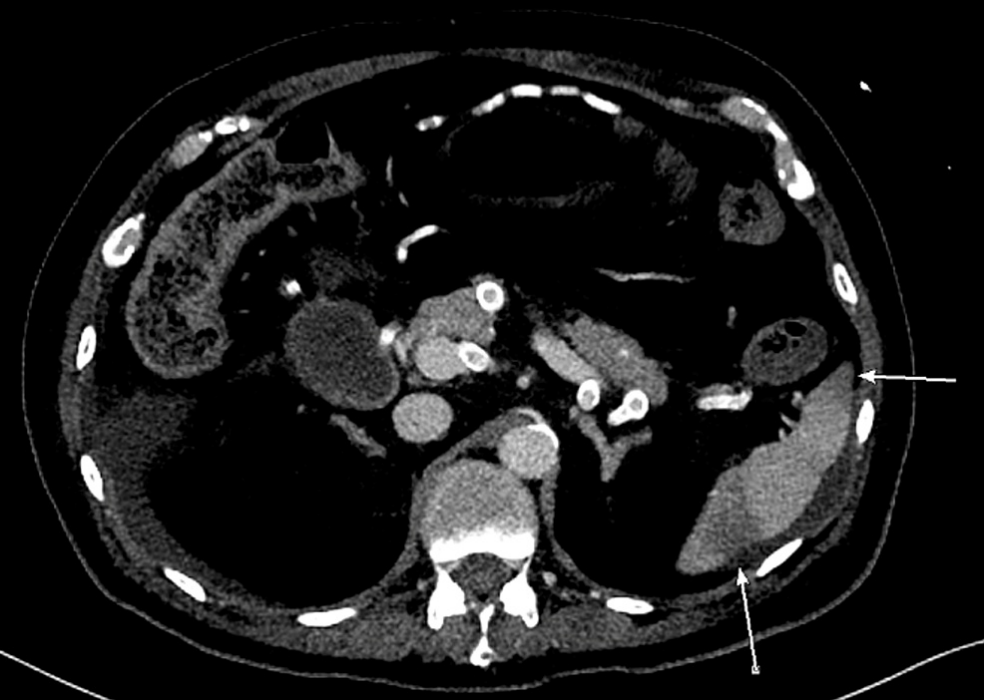
An axial plane image of the CT abdomen and pelvis showing the multiple splenic infarctions as demonstrated by the arrows and hypodensities within the spleen.

**Figure 2:**
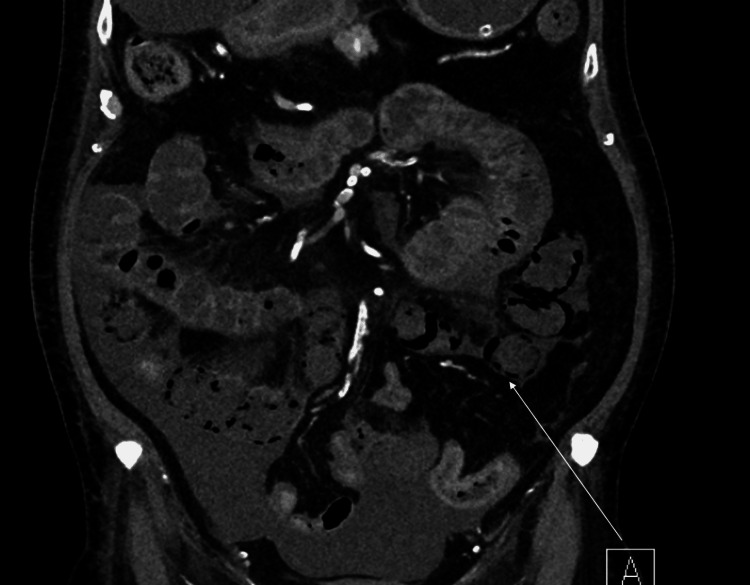
A coronal plane image of the CT abdomen and pelvis demonstrating jejunum and ileum wall hypoenhancement in comparison to the rest of the bowel, as well as pneumatosis intestinalis of jejunum and ileum as shown by the arrow.

Moreover, a positive blood culture from peripheral lines isolated *Klebsiella pneumoniae*. A CT scan of the abdomen and pelvis was obtained to investigate the cause of abdominal distension, which showed multiple splenic infarctions and bowel ischemia as shown in Figure 1 and Figure 2.

Consequently, the patient was admitted to the ICU as a case of septic shock secondary to acute mesenteric ischemia. Started on a combination of vancomycin and meropenem as empirical antibiotics. Micafungin was added a day later.

He underwent three exploratory laparotomies to resect ischemic bowel segments due to ongoing ischemia. In the last exploratory laparotomy, closure of the abdomen with end Ileostomy and mucous fistula formation was achieved. Postoperatively, a repeated set of blood cultures was sent.

Blood cultures grew yeast from the central line first then the peripheral line, while the patient was on micafungin, which was identified as *S. Capitata* by matrix-assisted laser desorption/ionization time-of-flight (MALDI-TOF) mass spectrometry. As a result, echinocandin was discontinued, and liposomal amphotericin B infusion (5 ml/kg) was initiated. Subsequently, IV voriconazole 250 mg every 12 hours was introduced six days later due to an observed inadequate therapeutic response. Despite an extensive workup, no apparent cause for the yeast infection was identified apart from the central line and possible intra-abdominal infection. The ophthalmologist's assessment for fungemia did not reveal any sign of endophthalmitis. A transesophageal echocardiogram confirmed the absence of vegetation. Results were obtained for the minimum inhibitory concentration (MIC) of the antifungal agents for *S. capitata* while the patient was on echinocandins, shown in Table 2. 

**Table 2:** Another susceptibility pattern was acquired when the patient was switched to liposomal amphotericin B due to the persistently positive blood cultures, in which the MIC for liposomal amphotericin B increased to 1 and the MIC for micafungin decreased to 4. MIC: minimum inhibitory concentration

Drug	Minimum Inhibitory Concentration (MIC)
Amphotericin B	0.5
Anidulafungin	2
Caspofungin	>8
Fluconazole	8
Micafungin	>8
Voriconazole	0.25

Twenty days after admission, the patient's health deteriorated significantly, leading to myocardial infarction, brain ischemia, abdominal infections, and ultimately passing due to multi-organ failure triggered by severe septic shock, likely stemming from the fungemia.

## Discussion

*S. capitata*, or *Magnusiomyces capitatus* ( formerly known as *Trichosporon capitatum* and *Blastoschizomyces capitatus*), is classified as an Ascomycota due to its cell structure and tendency to produce multiple arthroconidia. It utilizes breached skin and mucosal barriers to colonize immunocompromised patients leading to fatal clinical outcomes [1]. It is noteworthy that the only case report in Saudi Arabia, published by Ahmed et al., documenting an immunocompetent patient contracting *S. capitata*, underscore the rarity of such occurrences in this population [2]. Despite its infrequency, there is a notable global escalation in the incidence of this infection, emphasizing the need for heightened awareness and understanding of effective management strategies. Lo Cascio et al. reported several documented outbreaks in medical centers worldwide, particularly in hematology and ICU wards, highlighting the potential nosocomial sources of this infection [3]. In our case the patient’s blood cultures grew *S. capitata* after 72 hours of admission, raising the possibility of nosocomial acquisition, although no further cases or outbreaks were identified.

According to a study by Schuermans et al., *S. capitata* has an extremely high potential to cause disseminated and invasive diseases, especially in vulnerable hosts [4]. Myelosuppression in patients with hematological malignancies predisposes them to emerging opportunistic fungi like *S. capitata* to colonize and spread. Other factors like prolonged neutropenia, broad-spectrum antibiotic use, ICU hospital stay, organ transplantation, and aggressive chemotherapy damp down the body’s natural defense mechanisms making it favorable for *S. capitata* to thrive. The 30-day mortality associated with invasive diseases was estimated to be 60%, primarily due to breakthrough infections in patients treated with echinocandins [5]. In our patient, his blood cultures grew positive yeast multiple times while being empirically treated with echinocandins since *S. capitata* is intrinsically resistant to echinocandins and highly resistant to fluconazole [6,7].

Disseminated infections cause a wide spectrum of variable clinical presentations depending on the site of infection. Patients with pulmonary disease can present with dyspnea, cough, and sputum production with diffuse bilateral infiltrates and pleural effusion on a chest x-ray [7]. Patients with intra-abdominal involvement present with changes in bowel habits, abdominal pain, and jaundice, and rarely progress to peritonitis and abdominal compartment syndrome. Bladder and renal involvements mainly present with dysuria and hematuria. Other clinical manifestations include mental state changes and seizures with CNS involvement, skin lesions, and wound infections [7]. Our patient had disseminated fungemia, where his central line, blood, intra-abdominal fluid, endotracheal aspirate, and wound cultures were all positive for *S. capitata*. Despite the non-neutropenic status of our patient, the multiple sources of infections were tremendously difficult to control. As a result, the patient deteriorated rapidly and progressed to irreversible end-organ damage. 

Culturing blood or other sterile affected sites is necessary to diagnose *S. capitata*. Cultures typically take up to five days or more, which can lead to diagnostic and therapeutic delays. Microscopically, *Saprochaete* species are characterized by pseudo-hyphae and arthroconidia. MALDI-TOF mass spectrometry is the most accurate and superior device in detecting these species, allowing rapid identification [8].

*S. capitata* has a unique mutation on the *FKS* gene, which induces intrinsic resistance to echinocandins. As a result, breakthrough infections are often common due to the rarity of the species and the delay in diagnosis [7]. According to guidelines developed by the European Confederation for Medical Mycology (ECMM) and the European Society for Clinical Microbiology and Infectious Diseases (ESCMID), recommendations based on treatment response have been noted following amphotericin B formulation with or without flucytosine treatment and voriconazole. The use of echinocandins might be associated with worse outcomes. Where an amphotericin B formulation is used, liposomal amphotericin B has been used successfully [8]. We should keep in mind there are no comparative antifungal treatment trials for *S. capitata* infection and the recommendation is mainly based on in-vitro susceptibility results which highlights the importance of integrating the clinical presentation along with the results of MIC and outcome. Chen et al. demonstrated that *S. capitata* has a MIC of ≤ 1 mg/L to itraconazole, posaconazole, voriconazole, and isavuconazole, while MIC to ​​fluconazole is typically 16-32 mg/L although some strains have lower MICs [9]. Thus, the results of MIC of antifungals need to be interpreted with caution, as it can aid in selecting the appropriate treatment option to eradicate the infection. Based on our lab’s MIC results, we managed to tailor therapy to target *S. capitata*.

Cut-off values and clinical interpretive breakpoints have been developed for a number of antifungal agents and common fungal infections like Candida. However, less-common species like in our case, for which susceptibility data are limited, are increasingly reported in high-risk patients and breakthrough infections. In a large study done from 2002 to 2016 comparing MIC distributions for amphotericin B, fluconazole, itraconazole, voriconazole, flucytosine, and anidulafungin for 35 uncommon pathogenic yeast species determined using the Clinical and Laboratory Standards Institute (CLSI) broth microdilution method, 59 cases of *M. capitatus *were isolated. The study shows that the most effective antifungals are amphotericin-B and itraconazole (with or without 5FC). The percentages of isolates with resistant MIC (above the tentative breakpoints) are 2.4% for amphotericin-B, 2.9% for Itraconazole, and 0% for 5FC (the last is usually used as combination therapy in rare cases). On the other hand, 12.5% of isolates had MIC resistant to voriconazole and 72% of the isolates had MIC resistant to fluconazole (8 mg/L or above). As for echinocandin, anidulafungin was the only tested one with a 94% resistance rate, which is expected as it is known to be intrinsically resistant to echinocandin [10].

Similar to our case, there were two case reports of *S. capitata* infection in immunocompetent patients with intra-abdominal involvement necessitating laparotomies to control sources of infection. The first case was by Rahali et al., where the patient was in the same age group as the patient in the current case, with disseminated fungemia and a very poor outcome. The patient passed away unfortunately five days into admission [11]. In the second case, the patient was much younger and had isolated intraperitoneal positive cultures identifying *S. capitata* without disseminated diseases, which were all favorable to the outcome, and the patient, fortunately, survived [12]. However, given our patient’s complex hospital course, pre-existing comorbidities, severe multi-organ damage, and disseminated intravascular coagulation, there was no adequate therapeutic response even with targeted therapy.

## Conclusions

This case depicts *S. capitata* fungemia in an immunocompetent patient with a fatal outcome, which is a rather unusual target for this pathogen. The presence of multiple comorbidities that were poorly controlled accelerated the dissemination of the infection to involve multiple organs making it difficult to control. Prompt detection of *S. capitata* and initiation of proper antifungals like amphotericin B and voriconazole could improve outcomes in patients.
